# A radiomics nomogram for the ultrasound-based evaluation of central cervical lymph node metastasis in papillary thyroid carcinoma

**DOI:** 10.3389/fendo.2022.1064434

**Published:** 2022-11-30

**Authors:** Quan Wen, Zhixiang Wang, Alberto Traverso, Yujiang Liu, Ruifang Xu, Ying Feng, Linxue Qian

**Affiliations:** ^1^ Department of Ultrasound, Beijing Friendship Hospital, Capital Medical University, Beijing, China; ^2^ Department of Radiation Oncology (Maastro), GROW-School for Oncology and Reproduction, Maastricht University Medical Centre+, Maastricht, Netherlands

**Keywords:** nomogram, papillary thyroid carcinoma, ultrasound, lymph node metastasis, radiomics

## Abstract

**Purpose:**

To develop and validate a radiomics nomogram based on ultrasound (US) to predict central cervical lymph node (LN) metastasis in patients with papillary thyroid carcinoma (PTC).

**Methods:**

PTC patients with pathologically confirmed presence or absence of central cervical LN metastasis in our hospital between March 2021 and November 2021 were enrolled as the training cohort. Radiomics features were extracted from the preoperative US images, and a radiomics signature was constructed. Univariate and multivariate logistic regression analyses were used to screen out the independent risk factors, and a radiomics nomogram was established. The performance of the model was verified in the independent test cohort of PTC patients who underwent thyroidectomy and cervical LN dissection in our hospital from December 2021 to March 2022.

**Results:**

In the independent test cohort, the radiomics model based on long-axis cross-section and short-axis cross-section images outperformed the radiomics models based on either one of these sections (the area under the curve (AUC), 0.69 vs. 0.62 and 0.66). The radiomics signature consisted of 4 selected features. The US radiomics nomogram included the radiomics signature, age, gender, BRAF V600E mutation status, and extrathyroidal extension (ETE) status. In the independent test cohort, the AUC of the receiver operating curve(ROC) of this nomogram was 0.76, outperformingthe clinical model and the radiomics model (0.63 and 0.69, respectively), and also much better than preoperative US examination (AUC, 0.60). Decision curve analysis indicated that the radiomics nomogram was clinically useful.

**Conclusions:**

This study presents an efficient and useful US radiomics nomogram that can provide comprehensive information to assist clinicians in the individualized preoperative prediction of central cervical LN metastasis in PTC patients.

## Introduction

Thyroid carcinoma is one of the most common endocrine malignancies with a significantly increasing incidence in the last decade worldwide. Papillary thyroid carcinoma (PTC) accounts for more than 90% of patients with thyroid carcinoma, which has a stable high survival rate (1–5). However, cervical lymph node (LN) metastasis is prone to present in 40%-90% of PTC patients (6). The central cervical lymph nodes (LNs) are the sentinels of thyroid carcinoma metastasis and represent an important risk factor for recurrence in PTC patients (7–9). In recent years, the trend of surgical treatment has been that patients with PTC ≤ 1 cm in diameter without LN metastasis and extrathyroidal extension (ETE) can be treated conservatively (10). According to the revised American Thyroid Association (ATA) guidelines, lobectomy alone can be performed in patients without LN metastasis (11). Therefore, preoperative assessment for the presence or absence of LN metastasis remains an important issue. It may influence not only the staging of PTC but also its treatment.

High-frequency ultrasound (US) can be the recommended method to evaluate cervical LN status (11). Although the specificity of US is high in the diagnosis of cervical LN metastasis, the sensitivity is relatively low, especially in the diagnosis of central cervical LN metastasis (12–16). Therefore, prophylactic dissection of central cervical LN is currently performed in surgical treatment of many PTC patients (17). However, this kind of surgical treatment for PTC patients increases the incidence of surgical complications such as hypocalcaemia and recurrent laryngeal nerve injury (18, 19).

Previous studies have suggested that US features of thyroid carcinoma are predictors of cervical LN metastasis status (20–22). However, there are large differences in the results of these studies. Radiomics is based on extracting a large subset of quantitative features from medical images (23). This method has mainly been applied to computed tomography(CT) and magnetic resonance imaging (MRI) and has proven to be an effective method of differential diagnosis and predict the prognosis of many diseases since 2012 (24–26). In recent years, some clinical studies based on US radiomics have also been carried out in some diseases, and they have yielded better results than conventional US examination (27, 28).

A nomogram is a useful graphic method to visualize the probability of risk factors in a clinical event. There have been some previous studies using nomograms involving clinical risk factors and US radiomics to predict cervical LN metastasis (29, 30). However, some of these studies had small sample sizes or relied only on US radiomics and clinical features, lacking information of gene mutations associated with PTC aggressiveness, such as BRAF V600E mutation status. In the present study, we aimed to establish a predictive model based on risk factors, including demographic information, biomarker information, radiomics and macroscopic US characteristics, and we assessed the performance of this model in the individual preoperative prediction of central cervical LN metastasis in PTC patients.

## Materials and methods

### Patients

**Figure 1:**
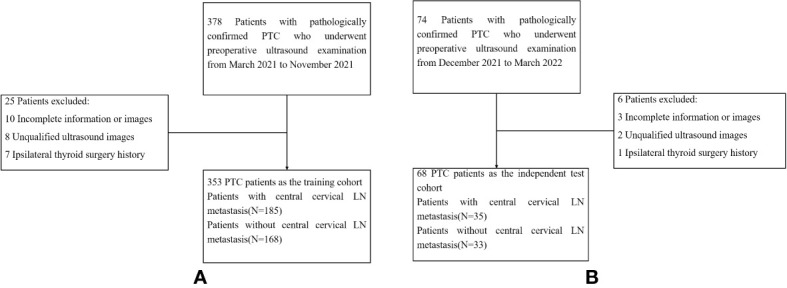
Flow-chart of patients selection. **(A)** showed the the enrollment process of patients in the training cohort. **(B)** showed the the enrollment process of patients in the test cohort. *LN,lymph node; PTC,papillary thyroid carcinoma*.

This study was approved by the Ethics Committee at the Affiliated Beijing Friendship Hospital of Capital Medical University, and the requirement for informed consent was waived. Patients who were diagnosed with suspicious PTC by preoperative fine needle aspiration (FNA) cytological results and received thyroidectomy and cervical LN dissection in our hospital between March 2021 and March 2022 were selected. **Figure 1** showed the enrolment procedure.

### Inclusion criteria

(1) All the patients received preoperative US examination on thyroid lesions and cervical LNs, preserving clear and complete DICOM-format image data; (2) the imaging data of the target lesions consisted of the long-axis cross-section and the short-axis cross-section; (3) thyroidectomy and cervical LN dissection were performed; (4) the postoperative pathological diagnosis of all the patients was PTC; and (5) the clinical information of these patients (including thyroid hormones and BRAF V600E mutation test results) was complete.

### Exclusion criteria

(1) Interventional therapy (such as microwave and radiofrequency therapies) or surgery on the thyroid was performed; (2) head and neck radiotherapy was performed; (3) postoperative pathological diagnosis of lesions identified them as non-PTC tumours, such as follicular carcinomas, medullary carcinomas, anaplastic carcinomas and metastatic carcinomas; (4) the US images were not clear and complete or did not meet the requirements above; and (5) the clinical information was incomplete.

### US image acquisition

All patients underwent a routine preoperative US examination on the thyroid lesions and cervical LNs, conducted by using a 5-12 MHz linear array transducer (iU22, Philips Medical Systems, Bothell, Wash). The patient was in a supine position with a pillow under the neck. The neck region was exposed as much as possible to perform the US examination on the thyroid lesions and cervical LNs using continuous scanning. The images were preserved as DICOM files. The US features were observed by US physicians with more than 10 years’ experience in thyroid US examination, and features that may be related to LN metastasis were recorded as follows: thyroid tumour size, tumour number, tumour echo pattern, tumour calcification, tumour vascularization and ETE. Assessment of cervical LN status was also performed by US physicians. Preoperative ultrasound examination was performed 1 week before surgery.

### Clinical information

Patients with PTC who received thyroidectomy cervical LN dissection in our hospital between March 2021 and November 2021 were enrolled in the training cohort. In addition, patients who received surgery were enrolled in the independent testing cohort between December 2021 and March 2022. The independent testing cohort was used to assess the generalizability of the predictive model.

The clinical information, including demographic information (age and gender), thyroid-stimulating hormone (TSH), thyroglobulin antibody (TgAb), thyroid peroxidase antibody (TPOAb) and BRAF V600E mutation status, were obtained from medical records. Preoperative TSH, TgAb, and TPOAb tests were performed 1 week before surgery. The FNA cytological examination showed category according to the Bethesda system.

### Surgery and pathology

All patients whose FNA cytological reports showed suspicious PTC underwent lobectomy and isthmectomy or total thyroidectomy with prophylactic cervical LN dissection. In addition, lateral LN dissection was performed in patients with suspicious lateral LN metastasis reported by preoperative FNA cytological examination or an intraoperative frozen pathological result. The surgically removed thyroid tissue and cervical LNs were subjected to pathological examination, and LN metastasis was confirmed. The LN status postoperative pathological report of the patients was used as the gold standard in the present study. The patients were divided into two groups (central cervical LN metastasis positive and negative groups) based on the postoperative pathological reports in the training cohort and independent testing cohort.

### Region-of-interest segmentation and radiomics feature extraction

To identify the lesions of the thyroid gland, ROIs were manually drawn on US images by US physicians with more than 10 years’ experience in thyroid US examination using the open-source software MRIcroGL v1.0 (http://www.mccauslandcenter.sc.edu/mricrogl/). The ROI was drawn on the solid component of the tumour, avoiding haemorrhagic, necrotic, and cystic areas.

US features were extracted from these ROIs on the long-axis cross-section and the short-axis cross-section of US images using PyRadiomics (version 3.0.1). The configuration file for PyRadiomics and the source codes are available on GitLab at https://gitlab.com/w654053334/Thyroid_Radiomics.

### Data preprocessing, feature selection and radiomics model development

For data preprocessing, radiomics features were normalized with the min-max normalization method both in the training cohort and the independent testing cohort. Second, we applied the K-nearest neighbour (KNN)-based missing value padding method, which can predict the probable value according to the K nearest neighbour data, to replace the missing data in the training cohort.

A feature-selection method was performed to select the most effective radiomics features. First, the independent sample t test was used to select features related to central cervical LN metastasis status from 208 features to remove nonsignificant features with *p >*0.05. Second, the optimal features were obtained from these 208 features with the least absolute shrinkage and selection operator (LASSO). After dimensionality reduction, 4 features were selected:

Horizontal: *Maximum2DDiameterColumn* (Shape), *ZoneEntropy* (Glszm).

Vertical: *ZoneEntropy* (Glszm), *RunLengthNonUniformity (*Glrlm*)*

The radiomics signature was obtained by the selected features with a nonzero coefficient weight of LASSO regression.

### Clinical and ultrasonic features selection, establishment of clinical model and clinical combined with US model

Univariate analyses and multivariate logistic regression were used to select the clinical risk factors (age, gender, TSH, TgAb, TPOAb and BRAF V600E mutation status) related to central cervical LN metastasis to generate the clinical model. Similarly, clinical risk factors and ultrasonic risk factors (tumour size, tumour number, tumour echo pattern, tumour calcification, tumour vascularization and ETE) were combined by logistic regression. The clinical combined with the US model was generated based on independent predictors of clinical and ultrasonic risk factors.

### Establishment of radiomic-related models and development of a US radiomics nomogram predictive model

After fully considering and comparing the results of the independent predictors of clinical and ultrasonic risk factors as well as the selected radiomics features, an improved model was generated by multivariable logistic regression. A clinical combined radiomics model was also developed for comparison with the improved model. An US radiomics nomogram based on the improved model was performed in the training cohort.

### Assessing the performance of the US radiomics nomogram predictive model

The performance of the US radiomics nomogram was assessed using a calibration curve. The discrimination performance of the radiomics nomogram was evaluated by the area under the curve (AUC) of the receiver operating characteristic (ROC) curve, sensitivity, and specificity. Then, the performance of the US radiomics nomogram was tested in independent testing cohorts. Decision curve analysis (DCA) was employed to determine the clinical usefulness of the US radiomics nomogram by the net benefits at different threshold values in the independent testing cohort.

### Statistical analysis

Statistical analyses were conducted by R software (version 3.6.1, https://www.r-project.org). Pearson’s chi-square test was used to compare differences for categorical variables. The independent sample t test was conducted for continuous variables with a normal distribution, whereas the Mann−Whitney U test was used for continuous variables without a normal distribution. All statistical significance levels were two-sided, with *p*< 0.05. LASSO feature selection, model development, calibration curve, decision curve and the nomogram were performed by R language. t test feature selection and logistic regression were performed in Python 3.8.

## Results

### Clinical characteristics

**Figure 2:**
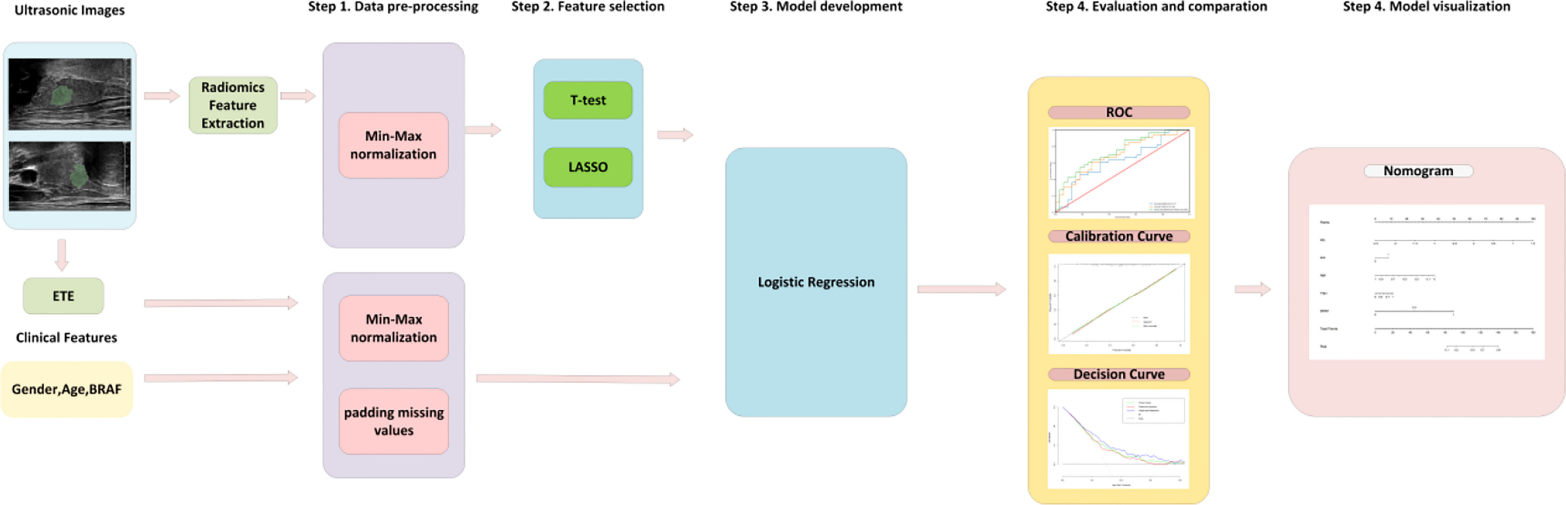
The flowchart for model development, evaluation and visualization. First, the features were processed by normalization and padding the missing values. Second, radiomics features were selected by T test and the Lasso algorithm. Then, the logistic regression model was trained in the training cohort. Finally, the model was evaluated in the independent test cohort and summarized as a nomogram. *ETE, extrathyroidal extension; BRAF, BRAF V600E mutation; LASSO, least absolute shrinkage and selection operator; ROC, receiver operating curves*.

The study flowchart is shown in **Figure 2**. A total of 353 patients with PTC were enrolled in the training cohort with an average age of 44.04 ± 11.69 years (range: 20–74 years) and a male-to-female ratio of 93:260. In addition, 68 patients with PTC were enrolled in the independent testing cohort with an average age of 41.75 ± 10.09 years (range: 26–67 years) and a male-to-female ratio of 14:54. The clinical characteristics of patients in the training and independent test cohorts are shown in **Table 1**. Except for BRAF V600E mutation status, there was no significant difference between the two cohorts in demographic information, clinical information, pathological reports, or US image features (*p* > 0.05). The rates of central cervical LN metastasis were 52.4% (185/353) and 51.5% (35/68) in the training and independent test cohorts, respectively, and there were no significant differences between them (*p* = 0.887). However, the sensitivity of US-reported LN status by preoperative US examination was 28.6% (53/185) and 20.0% (7/35) in the training and independent test cohorts, respectively, in our study.

**Table 1:** Patient characteristics of the training cohort and the independent testing cohort.

Characteristic	Training cohort (n = 353)	Independent testing cohort (n = 68)	P
Age, mean ± SD, years	44.04 ± 11.69	41.75 ± 10.09	0.132
<45 years	191	45	0.066
>=45 years	162	23	
Gender			
Male	93	14	0.318
Female	260	54	
TSH, No. (%)			
<4.94 ng/ml	335	64	1.000
>=4.94 ng/ml)	18	4	
TGAB, No. (%)			
<4.11 IU/ml	280	53	0.798
>=4.11 IU/ml	73	15	
TPOAB, No. (%)			
<5.61 IU/ml	268	48	0.352
>=5.61 IU/ml	85	20	
Internal echo pattern			
Uniform	350	66	0.397
Nonuniform	3	2	
ETE			
Positive	209	35	0.237
Negtive	144	33	
Tumor size	1.11 ± 0.66	0.98 ± 0.57	0.138
<10mm	183	41	0.201
>=10mm	170	27	
Multifocality			
Positive	122	30	0.133
Negtive	231	38	
Tumor calcification			
Positive	261	51	0.855
Negtive	92	17	
Tumor vascularization			
Without	162	35	0.682
Rare	171	30	
Abundant	20	3	
US reported LN status, No. (%)			
Positive	68	7	0.077
Negtive	285	61	
BRAF V600E			
Positive	349	53	0.000
Negtive	4	15	
Postoperative pathological result reported LN status, No. (%)			
Positive	185	35	0.887
Negtive	168	33	

SD, standard deviation; ETE, extrathyroidal extension; TSH, thyroid-stimulating hormone, TgAb, thyroglobulin antibody, TPOAb, thyroid peroxidase antibody; US, ultrasound; LN, lymph node.

### Establishment of US radiomics signature

**Figure 3:**
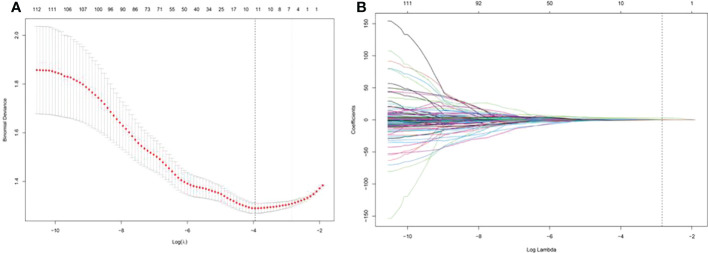
Least absolute shrinkage and selection operator (LASSO) screening process for feature selection. **(A)** was used to reduce the dimension of the grouping characteristics **(B)**. Four features corresponded to the minimum error.

For each nodule, one US image of the long-axis cross-section and one US image of the short-axis-cross-section were used for analysis. For patients with more than one nodule, the images of all suspected nodules were applied. A total of 208 imaging features were extracted from ROIs of US images in each nodule in the long-axis cross-section and the short-axis cross-section. A total of 117 radiomics features with no statistical significance in the training cohort according to the t test were eliminated. Then, using LASSO regression and 10-fold cross-validation, 4 features with nonzero coefficients were selected in the training cohort (**Figures 3A, B**). According to the results of the LASSO regression analysis, the mathematical expression of the radiomics signature was:


$$\begin{array}{l} \text{Radiomics signature}\\ =-1.668+0.003\times \text{original}\_\text{shape}\_\text{Maximum}2\text{DDiameterColumn}(\text{Horizontal})\\ +\;0.009\times \text{original}\_\text{glszm}\_\text{ZoneEntropy}(\text{Horizontal})\\ +\;0.165\times \text{original}\_\text{glszm}\_\text{ZoneEntropy}(\text{Vertical})\\ +\;0.001\times \text{original}\_\text{glrlm}\_\text{RunLengthNonUniformity}(\text{Vertical}) \end{array}$$

The AUC of US radiomics features was 0.69 (95% CI: 0.57-0.80) in the independent test cohort.

### The performance of different models to predict central cervical LN metastasis in PTC patients

The predictive performance of the radiomics model based on the images of the long-axis-cross section combined with the short-axis-cross section was superior to that of the radiomics model based on the image of either one of these sections in the independent test cohort (AUC, 0.69 vs. 0.62, 0.66). The specificity of the radiomics model based on these two sections was much higher than that of the model based on a single section (0.85 vs. 0.64, 0.56) (**Table 2**).

**Table 2:** The performance of radiomics models to predict central cervical LN metastasis of PTC based on extracted features in different sections.

Models	Short Axis Cross Section	Long Axis Cross Section	Short Axis Cross Section+ Long Axis Cross Section
AUC	0.66 (95%CI: 0.58-0.74)	0.62 (95%CI: 0.55-0.70)	0.69 (95%CI: 0.57-0.80)
SEN	0.60 (95%CI: 0.41-0.80)	0.63 (95%CI: 0.42-0.85)	0.49 (95%CI: 0.31-0.66)
SPE	0.64 (95%CI: 0.41-0.87)	0.56 (95%CI: 0.33-0.78)	0.85 (95%CI: 0.68-0.95)

AUC, area under curve; SEN, sensitivity; SPE, specificity; CI, confidence interval; LN, lymph node; PTC, papillary thyroid carcinoma.

Age, gender and BRAF V600E mutation status were identified as independent clinical predictors of central cervical LN metastasis in PTC patients by multivariate logistic regression, based on which the clinical model was developed. The tumour size, tumour Internal echo pattern and ETE were identified as ultrasonic independent predictors of central cervical LN metastasis in PTC patients by multivariate logistic regression. The clinical combined with US model was developed based on clinical and ultrasonic risk factors (age, gender, BRAF V600E mutation status, tumour size, tumour internal echo pattern and ETE). The clinical combined with the radiomics model was developed based on clinical risk factors (age, gender and BRAF V600E mutation status) and radiomics radiomics signature. Considering that the selected radiomics features cannot reflect the ETE status of PTC, an improved model, the clinical combined with radiomics and ETE model, was developed based on clinical risk factors (age, gender and BRAF V600E mutation status), radiomics signature and ETE status in US features.

**Figure 4:**
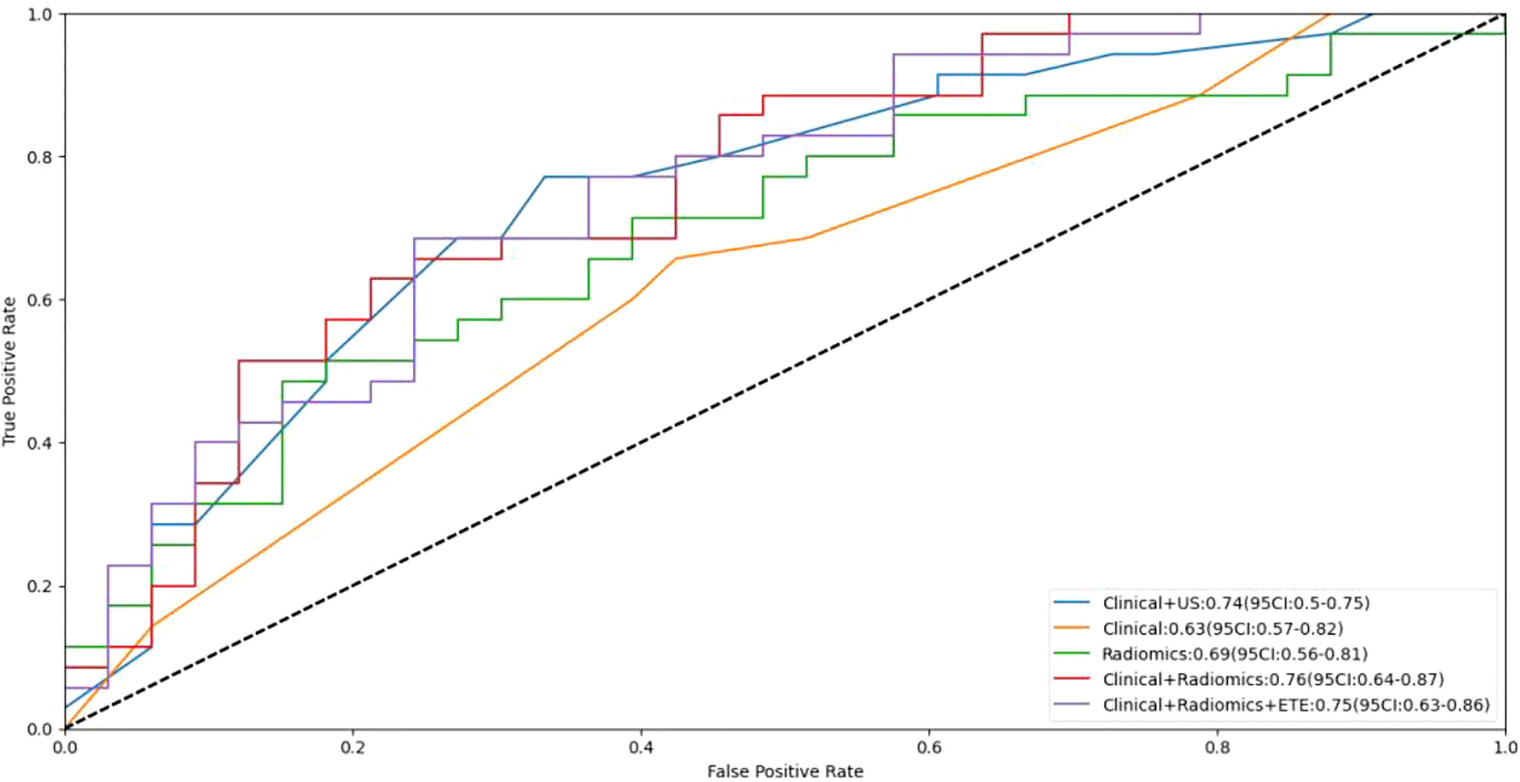
Receiver operating curves (ROC) for the models. *US, ultrasound; ETE, extrathyroidal extension*.

The AUCs of the clinical combined with US model, the clinical combined with radiomics model, and the clinical combined with radiomics and ETE model were 0.75, 0.76 and 0.75, respectively, in the independent test cohort. There were no significant differences between any two of them (*p* > 0.05). The performance of these three models was better than that of the clinical and radiomics model (AUC, 0.63 and 0.69, respectively). The sensitivity of the clinical combined with US model, the clinical combined with radiomics model, and the clinical combined with radiomics and ETE model were 0.77, 0.63 and 0.69, respectively. The specificity of these three models were 0.67, 0.79, and 0.76, respectively. In the independent test cohort, the AUC, sensitivity, and specificity of preoperative US in the diagnosis of central cervical LN metastasis were 0.60, 0.20, and 1.00, respectively (**Table 3** and **Figure 4**).

**Table 3:** The performance of different models to predict central LN metastasis of PTC based on clinical, ultrasound and radiomics feasure.

Models	Clinical	Clinical+US	Clinical+Radiomics	Clinical+Radiomics+ETE
AUC	0.63 (95%CI: 0.56-0.81)	0.74 (95%CI: 0.50-0.75)	0.76 (95%CI: 0.64-0.87)	0.75 (95%CI: 0.64-0.86)
SEN	0.66 (95%CI: 0.48-0.81)	0.77 (95%CI: 0.60-0.90)	0.63 (95%CI: 0.45-0.79)	0.69 (95%CI: 0.51-0.83)
SPE	0.58 (95%CI: 0.39-0.75)	0.67 (95%CI: 0.48-0.82)	0.79 (95%CI: 0.61-0.91)	0.76 (95%CI: 0.58-0.89)

AUC, area under curve; SEN, sensitivity; SPE, specificity; CI, confidence interval; US, ultrasound; ETE, extrathyroidal extension; PTC, papillary thyroid carcinoma.

**Figure 5:**
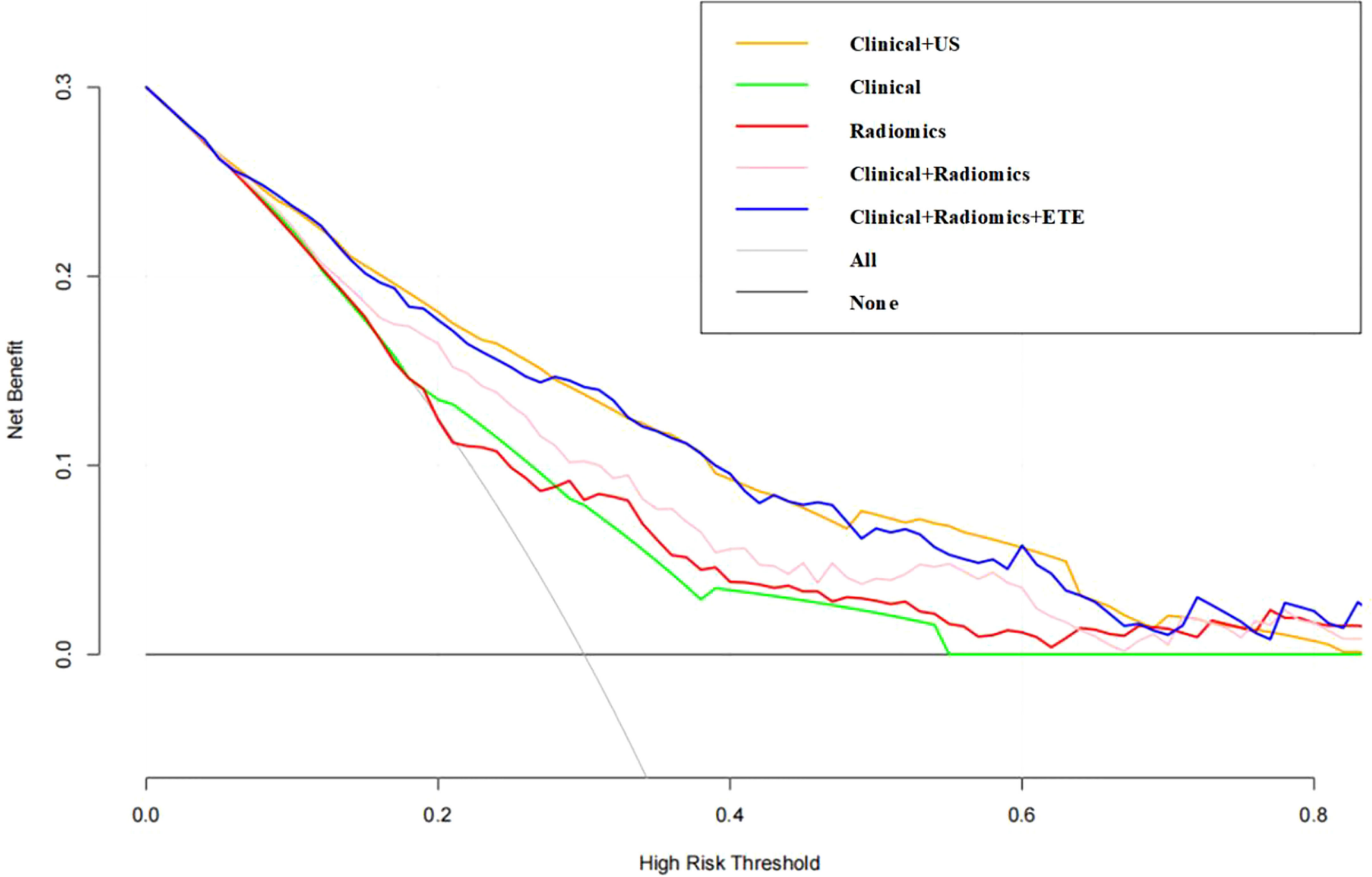
Decision curve of the nomogram. The y-axis indicates the net benefit; the x-axis indicates the threshold probability. *US, ultrasound; ETE, extrathyroidal extension*.

The DCA demonstrated that the clinical combined with radiomics and ETE model had the greatest overall net benefit among the tested models when the threshold probability for a clinician or a patient ranged from 0.1 to 0.8, and the clinical combined with radiomics and ETE model was more beneficial than either the treat-all or the treat-none strategy (**Figure 5**).

### Development and performance of the radiomic nomogram

**Figure 6:**
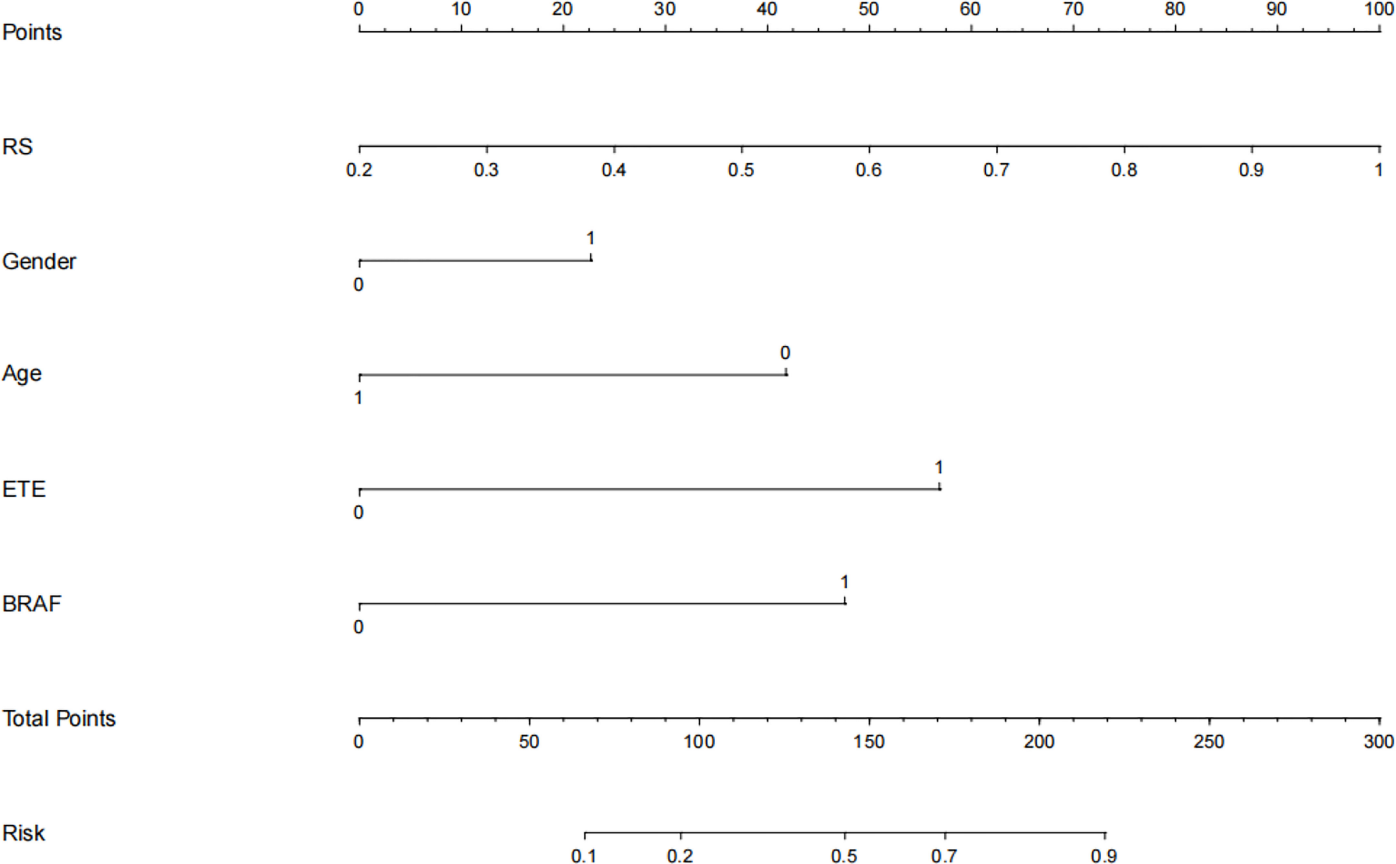
Nomogram visualization. The nomogram presents the developed model predicting central cervical lymph node(LN) function by radiomics signature (RS), gender, age, extrathyroidal extension (ETE), and BRAF V600E mutation (BRAF).

**Figure 7:**
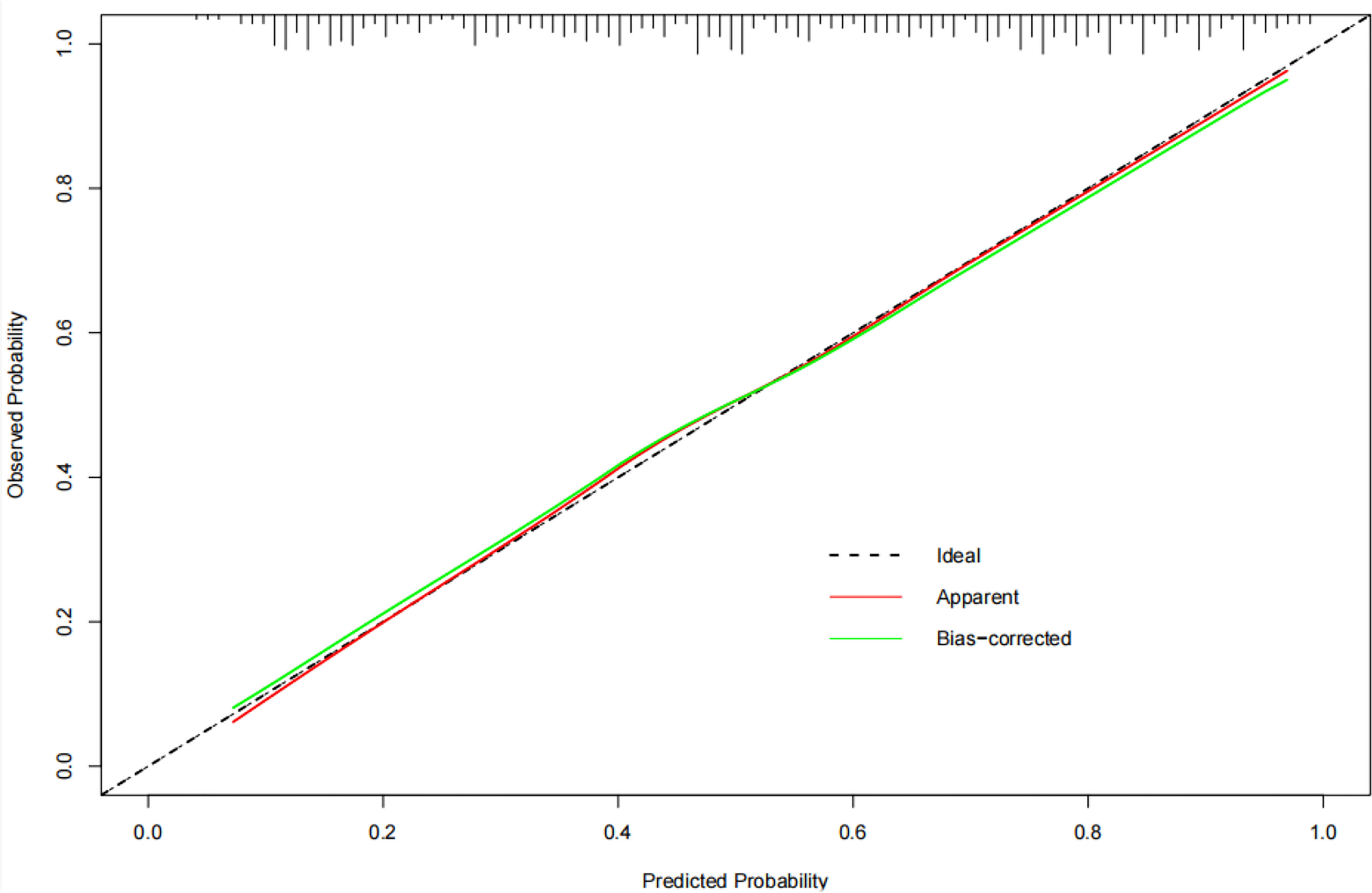
The calibration curve of the nomogram. The calibration curve shows whether the nomogram has goodness of fit. where the grey diagonal line is the ideal prediction. The closer to the diagonal line, the better the performance is.

Based on the above results, the clinical combined with radiomics and the ETE model and the clinical combined with the US model performed similarly well. However, in terms of guiding the selection of clinical treatment options in the future, surgeons have higher requirements for the specificity of the predictive model to avoid unnecessary central cervical LN dissection; on this basis, we chose the clinical combined with radiomics and ETE model to generate a nomogram. Therefore, a radiomics nomogram incorporating these five predictors (age, gender, BRAF V600E mutation status, radiomics signature and ETE) was developed (**Figure 6**). **Figure 7** shows the calibration curve of the nomogram. results showed good agreement between the prediction curve and standard curve. Thus, our nomogram performed well in the independent test.

## Discussion

Although PTC has a stable high survival rate, cervical LN metastasis is prone to exist at the early stage (4–6, 31). Local recurrence and distant metastasis are highly associated with cervical LN metastasis (7, 8, 32). According to the 2015 ATA guidelines, preoperative assessment for the presence of cervical LN metastasis and ETE is the premise of determining the reasonable surgical strategy for PTC (11). The sensitivity of diagnosing cervical LN metastasis by preoperative US examination is relatively low, especially for central cervical LN metastasis (12–15). In our study, only 28.6% (53/185) of central cervical LN metastasis were reported by preoperative US examination in the training cohort, while in the independent cohort, only 20.0% (7/35) of central cervical LN metastasis were reported. Therefore, prophylactic central cervical LN dissection is currently chosen by many surgeons (17). However, there is still controversy because of the increased risk of hypocalcaemia and recurrent laryngeal nerve injury (19).

Many previous studies have attempted to establish clinical predictive models for cervica LN metastasis in PTC patients. In addition, some available studies have suggested that there is a relationship between US features and cervica LN metastasis in PTC patients. Mao et al. found that age, gender, multifocality, tumour size and ETE were significantly associated with cervical LN metastasis after they performed a systematic review and meta-analysis of 21 related studies (33). Liu et al. showed that male gender, age ≤45 years, tumour size > 1.0 cm, ETE and US features of microcalcifications were independent risk factors for cervical LN metastasis (34). The AUC range of predictive models based on clinical risk factors combined with US features ranged from 0.742 to 0.799 (20–22). Preoperative US examination is often affected by the subjective factors of US physicians. The identification of US features by different US physicians may exhibit differences.

Radiomics is a more precise, objective, and efficient method that can improve conventional imaging diagnosis. Radiomics extracts hundreds of quantitative features from images, and self-training and learning are conducted based on the pathological results to assist in clinical diagnosis (24–28, 35). Available studies have been conducted to develop radiomics models based on CT or US images to predict cervical LN metastasis or lateral cervical LN metastasis in PTC (29, 36, 37). These results suggested that radiomics models help to improve the diagnostic accuracy of cervical LN metastasis. In the present study, US radiomics features were extracted from the training cohort on the long-axis cross section and the short-axis cross section. After data analysis, original_shape_Maximum2DDiameterColumn_x, original_glszm_ZoneEntropy_x, original_glszm_ZoneEntropy_y, and original_glrlm_RunLengthNonUniformity_y were related to central cervical LN metastasis in PTC. The maximum 2D diameter was used to evaluate the largest distance of the tumour in the scanning slices. original_glrlm_RunLengthNonUniformity measures the similarity of run lengths throughout the image. original_glszm_ZoneEntropy measures the randomness in the distribution of zone sizes and grey levels. original_glrlm_RunLengthNonUniformity and original_glszm_ZoneEntropy estimated the homogeneity of echoes of the tumour in the scanning slices. In the US features, tumour size and internal echo were also independent predictors in multivariate analysis. Therefore, there is a corresponding relationship between the US radiomics features and the US features. However, radiomics features mainly show the microscopic information of ROI, lacking information of adjacent anatomical structures. In the selected radiomics features, no features corresponded to ETE, which was also an independent predictor in US features of central cervical LN metastasis. ETE, as an important feature for central cervical LN, cannot be shown in the radiomics model. Therefore, we attempted to incorporate ETE into the predictive model to develop a clinical combined with radiomics and ETE. The AUC of the improved model showed no significant difference from that of the clinical combined with US model (0.75 vs. 0.74, *p* = 0.688), but the clinical combined with radiomics and ETE model was superior to the clinical combined with US model in terms of specificity (0.76 vs. 0.67). Although the AUC of the clinical combined with radiomics and ETE model showed no significant difference (0.75 vs. 0.76, *p* = 0.903) from that of the clinical combined with radiomics model, the sensitivity of the clinical combined with radiomics and ETE model was improved over that of the clinical combined with radiomics model (0.69 vs. 0.63). This result suggested that ETE is an important complement to radiomics features and is of great significance for improving the predictive value of central cervical LN metastasis in PTC. According to available studies, ETE was identified by US physicians in preoperative US examinations with a sensitivity of 30% and a specificity of 93% (38). Therefore, the predictive result of our models may be affected by the result of ETE identified by US examinations. In future studies, we will attempt to improve the accuracy of ETE identification by contrast-enhanced US or radiomics to optimize our model.

Some previous studies suggested that BRAF V600E was associated with the aggressiveness of PTC and poor prognosis. Zhang et al. found that the BRAF V600E mutation was also a predictive factor for cervical LN metastasis in addition to age, tumour size, microcalcification and nonconcomitant Hashimoto’s thyroiditis (39). To the best of our knowledge, there is no report that a US radiomics model involving BRAF V600E mutation status would be developed to predict central cervical LN metastasis in PTC patients. We speculated that available studies on machine learning–based radiomics may not fully reflect the central cervical LN metastasis status of PTC, even including some clinical indicators. Therefore, in this study, we compared 5 different predictive models, each of which involved BRAF V600E mutation status. In our study, there were significant differences between the training cohort and the independent testing cohort in BRAF V600E mutation status (*p* = 0.000). The reason was that the sample size included in this study was not large enough, the training cohort was from retrospective cases, and the independent testing cohort was from prospective cases, causing a bias that may have affected the study results. In this study, it was difficult to evaluate whether BRAF V600E mutation status was helpful to improve the predictive efficacy of models for central cervical LN metastasis in PTC. Further studies with larger sample sizes should be carried out.

To provide a useful tool for surgeons, we developed a radiomics nomogram based on the clinical combined with radiomics and ETE model. Our nomogram showed good discrimination and calibration in the independent test cohort. The AUC of the nomogram was 0.75 in the independent test cohort, had greater predictive efficacy than the clinical combined and radiomics models (0.63 and 0.69, respectively) and was much better than the preoperative US examination reported in central cervical LN metastasis (AUC, 0.60), which was conducted by US physicians with more than 10 years’s experience in thyroid ultrasonography. The fine adjustment of the image and the subjective factors of physicians have a great influence on the identification and judgement of the lesion characteristics. In our study, the clinical combined with radiomics and ETE model had equivalent predictive efficiency to the clinical combined with US model. However, applying the radiomics nomogram is more standardized and efficient. If this method is applied clinically, it can improve the efficiency and accuracy of physicians in diagnosing central cervical LN metastasis and can contribute to effectively avoiding unnecessary prophylactic central cervical LN dissection.

US is a dynamic examination, and a single section cannot fully reflect all the information of the lesion. In our study, the image features of the long-axis cross-section and the short-axis cross-section of each lesion were extracted, which made up for the disadvantage that the characteristics of this subsection are not obvious in a single section to a certain extent. In future studies, we will also build a radiomics model based on multislice US images to provide more information to improve predictive efficiency.

There were several limitations in our study. First, in this study, the sample size was limited, and it was from a single centre. The images were collected by the same ultrasound doctor using the same ultrasound machine. Therefore, some bias has affected the results of our study. In future studies, it will be necessary to carry out multicentre research, increase the sample size, and observe the stability and effectiveness of the model when different doctors operate different equipment. And we will establish a standardized image collection process. Second, contrast-enhanced US and elastography can play operative roles in predicting cervical lymph nodes metastasis (40, 41).However, our radiomics study extracted radiomics features only from conventional ultrasound images. In the future, we will attempt to extract radiomics features of elastography and contrast-enhanced US images and will develop a radiomics nomogram based on multimodal US images. Third, although BRAF V600E mutation status was not incorporated into our US radiomics nomogram, the correlation between BRAF V600E mutation and central cervical LN metastasis in PTC patients was not fully confirmed in our study, and we need to perform a multicentre study with a large sample size for further verification. We will also add other related genes that are likely associated with central cervical LN metastasis in PTC patients to our nomogram to improve the preoperative predictive ability for central cervical LN in patients with PTC.

## Conclusion

In conclusion, a predictive model was established based on the US radiomics signature and clinical and ultrasonic risk factors. Although the predictive value of the US radiomics nomogram was close to that of the clinical combined with US model, our nomogram is a more precise, objective and efficient method than the conventional US model. ETE was incorporated into our nomogram and made up for the shortage of radiomics, which mainly shows the microscopic information of the ROI, lacking information of adjacent anatomical structures. Therefore, this US radiomics nomogram can provide comprehensive information to assist clinicians in individually predicting central cervical LN metastasis in PTC patients.

## Data availability statement

The raw data supporting the conclusions of this article will be made available by the authors, without undue reservation.

## Ethics statement

The studies involving human participants were reviewed and approved by the Ethics Committee at the Beijing Friendship Hospital of Capital Medical University. Written informed consent for participation was not required for this study in accordance with the national legislation and the institutional requirements.

## Author contributions

All authors contributed to the study conception and design. Material preparation, data collection and analysis were performed by QW, YL, RX, ZW. The first draft of the manuscript was written by QW, ZW and all authors commented on previous versions of the manuscript. All authors contributed to the article and approved the submitted version.

## Acknowledgments

We thank the Chinese Scholarship Council (CSC) for their financial support for studying abroad.

## Conflict of interest

The authors declare that the research was conducted in the absence of any commercial or financial relationships that could be construed as a potential conflict of interest.

## Publisher’s note

All claims expressed in this article are solely those of the authors and do not necessarily represent those of their affiliated organizations, or those of the publisher, the editors and the reviewers. Any product that may be evaluated in this article, or claim that may be made by its manufacturer, is not guaranteed or endorsed by the publisher.
